# Ependymoma relapse goes along with a relatively stable epigenome, but a severely altered tumor morphology

**DOI:** 10.1111/bpa.12875

**Published:** 2020-07-28

**Authors:** Denise Yang, Till Holsten, Daniela Börnigen, Stephan Frank, Christian Mawrin, Markus Glatzel, Ulrich Schüller

**Affiliations:** ^1^ Department of Pediatric Hematology and Oncology University Medical Center Hamburg‐Eppendorf Hamburg Germany; ^2^ Research Institute Children's Cancer Center Hamburg Hamburg Germany; ^3^ Bioinformatics Core Unit University Medical Center Hamburg‐Eppendorf Hamburg Germany; ^4^ Division for Neuropathology University Hospital Basel Basel Switzerland; ^5^ Institute for Neuropathology University of Magdeburg Magdeburg Germany; ^6^ Institute of Neuropathology University Medical Center Hamburg‐Eppendorf Hamburg Germany

**Keywords:** ependymoma, methylation, morphology, relapse

## Abstract

The molecular biology of ependymomas is not well understood and this is particularly true for ependymoma relapses. We aimed at finding out if and to which extent, relapses differ from their corresponding primary tumors on the morphological, chromosomal and epigenetic level. We investigated 24 matched ependymoma primary and relapsed tumor samples and, as a first step, compared cell density, necrosis, vessel proliferation, Ki67 proliferative index, trimethylation at H3K27 and expression of CXorf67. For the investigation of global methylation profiles, we used public data in order to analyze copy number variation profiles, differential methylation, methylation status and fractions of hypo‐ and hypermethylated CpGs in different epigenomic substructures. Morphologically, we found a significant increase with relapse in cell density and proliferation. H3K27 trimethylation and CXorf67 expression remained stable between primary and relapse tumor samples, and the analysis of DNA methylation profiles neither revealed significant differences in copy number variations nor differentially methylated regions. Significant differences in the methylation status were found for CpG islands, but also in N Shelves or S Shelves, depending on the molecular subgroup. The fraction of probes changing their methylation in the epigenomic substructures appeared subgroup‐specific. Most changes occur in CpG islands, for which relapsed tumors demonstrate higher methylation values than primary tumors. The morphological differences reflect increased aggressiveness upon ependymoma relapse, but, despite slight changes, this observation does not appear to be sufficiently explained by epigenetic changes.

## Introduction

Ependymomas are neuroepithelial tumors of the central nervous system (CNS). They occur in the supratentorial or infratentorial region of the brain, but also in the spinal cord. According to the WHO classification of tumors of the CNS, ependymomas are classified into WHO grades I, II, or III (19). However, WHO grading is often difficult due to ill‐defined diagnostic criteria, intratumoral heterogeneity and inter‐observer variability. Especially, the distinction between grade II and grade III ependymomas is hardly reliable, which makes it difficult to use histopathological grading as a prognostic marker (7). Recently, nine distinct molecular subgroups were described based on global DNA methylation profiling with three subgroups in each of the three compartments (supratentorial region, posterior fossa and spinal cord). This new molecular classification correlates much better with clinical findings than histopathological classification, making it relevant for future clinical trials (23, 25). Later, posterior fossa ependymomas of group A (PF‐EPN‐A) were further classified into two major subgroups comprising nine underlying subtypes (24). In addition, spinal ependymomas with *MYCN* amplification were suggested to establish an own molecular subgroup (8). Ependymal tumors can affect every age group, and location as well as prognosis are strongly associated with age. Ependymomas occurring in adults are rather located in the spinal cord, whereas ependymomas occurring in children or adolescents are more likely to be found in the brain. Prognosis is relatively poor for children aged 0–19 years and patients aged 65+ (35). In a retrospective multi‐center analysis of 103 pediatric patients with WHO grade II/III intracranial ependymoma, 10‐year overall survival (OS) was 50 ± 5%, whereas progression‐free survival (PFS) was 29 ± 5%. Even a decade or more after diagnosis, OS and PFS decreased continuously, reflecting the poor long‐term outcomes of pediatric ependymoma. Relapses were almost all confirmed to be recurrent ependymomas instead of radiation‐induced secondary malignant gliomas and were most likely to occur at the primary site, with occasional spread to the spinal cord or other sites of the brain (20). These findings demonstrate that there is still a huge lack of appropriate therapy, especially when it comes to ependymoma relapses. Although relapse patterns and molecular biology of other brain tumors, such as medulloblastomas or glioblastomas (10, 16, 31), are already well investigated, there is only very limited insight into the molecular mechanisms of ependymoma progression and relapse. A recent study found methylation profiles of recurrent ependymomas to cluster into the same molecular subgroup as the corresponding primary tumor, indicating that molecular subgroups remain stable over time (25). However, further understanding of the histomorphology and molecular biology of ependymoma relapses is needed in order to establish effective therapy for ependymoma relapses, particularly in consideration of the new molecular classification (23, 25).

## Materials and Methods

### Samples

Tumor samples were contributed by the Institute of Neuropathology, University Medical Center Hamburg‐Eppendorf (n = 19), the Division of Neuropathology, University Hospital Basel (n = 4) and the Institute of Neuropathology, University of Magdeburg (n = 3). Inclusion criterion was the diagnosis of an ependymoma with at least one ependymoma relapse. Diagnosis of two cases turned out to be incorrect with one case being a papillary tumor of the pineal region and the other one being an *IDH*‐mutated glioma after careful reevaluation. Altogether, we investigated 24 primary ependymal tumors and 38 corresponding relapsed ependymal tumors. The use of biopsy‐specimens for research upon anonymization was in accordance with local ethical standards and regulations at the University Medical Center Hamburg‐Eppendorf.

Age at diagnosis ranged from 0.4 to 62.7 years. Molecular subtype was determined by DNA methylation profiling for cases #1, #10 and #21 and by panel sequencing for case #2. For cases #4, #16 and #17, DNA methylation profiling was performed, but did neither match to a specific molecular subtype of ependymoma nor to one of the previously described CNS tumor DNA methylation classes (4). Still, careful histological reevaluation confirmed the diagnosis of ependymoma. For the remaining posterior fossa ependymoma cases #5–9 and #11–15, molecular subtype was inferred from the H3K27 trimethylation status (26). For the remaining supratentorial ependymoma case (#3), the molecular subtype was deduced from p65 status (27). Additionally, we confirmed by FISH analysis that RELA break apart is present in the primary as well as in one relapsed ependymoma sample for case #2 and #3 (data not shown). Copy number profiling for spinal ependymoma case #19 revealed *MYCN* amplification. Thus, this case was classified as SP‐EPN‐MYCN. For the remaining spinal ependymomas, molecular subgroup was not determined. Cases #2, #7, #8, #10, #11, #12, #15, #16, #19, #23 have been tested for the presence of histone H3K27M mutation and turned out to be negative. Altogether, the cohort consists of four supratentorial ependymomas, hereof three ST‐EPN‐RELA and one tumor with no match to a known molecular subgroup, 13 posterior fossa tumors, hereof six PF‐EPN‐A, five PF‐EPN‐B and two tumors with no match to a known molecular subgroup, and seven spinal tumors, including one SP‐EPN‐MYCN ependymoma and one SP‐MPE ependymoma. Further epidemiological and clinical details of patients are summarized in Table 1. Tumor samples were obtained as formalin‐fixed, unstained tissue sections from paraffin blocks. H&E staining as well as immunohistochemistry using antibodies against Ki67, H3K27me3 and CXorf67 were performed according to standard protocols.

**Table 1:** Epidemiological and clinical data of all analyzed patients with ependymoma recurrences

Case #	Gender	Age of onset [years]	Tumor localization	WHO grade of primary tumor	Molecular subtype	Number of recurrences	Time to following biopsy [months]	Localization of relapse	WHO grade of relapse	Follow‐up [months]	Status
1	F	12.2	Supratentorial	III	ST‐EPN‐RELA	1	36.2	local	III	36.2	Alive
2	M	26.4	Supratentorial	III	ST‐EPN‐RELA	7	8.1, 5.5, 14.9, 3.3, 6.6, 2.3, 11.3	local, metastatic	III, III, III, III, III, III, III	122.0	Alive
3	M	51.7	Supratentorial	III	ST‐EPN‐RELA	1	31.8	Local	III	31.9	Alive
4	M	50.6	Supratentorial	III	none	1	15.7	Local	III	15.7	Alive
5	F	0.7	Posterior fossa	III	PF‐EPN‐A	2	31.3, 11.0	n.a.	III, III	42.3	Alive
6	M	1.3	Posterior fossa	III	PF‐EPN‐A	1	26.6	local	III	26.6	Alive
7	M	1.5	Posterior fossa	III	PF‐EPN‐A	1	95.2	local	III	103.8	Alive
8	F	3.1	Posterior fossa	III	PF‐EPN‐A	1	1.2	local	III	14.9	Alive
9	M	3.4	Posterior fossa	III	PF‐EPN‐A	2	12.1, n.a.	n.a.	n.a.	12.1	Alive
10	M	8.8	Posterior fossa	III	PF‐EPN‐A	3	17.7, 26.5, 4.9	local, metastatic	III, III, III	52.0	Alive
11	M	10.2	Posterior fossa	II	PF‐EPN‐B	2	12.0, 76.6	local	II, II	121.1	Alive
12	M	25.7	Posterior fossa	III	PF‐EPN‐B	2	68.2, 32.5	n.a.	III, III	127.0	Alive
13	M	53.3	Posterior fossa	II	PF‐EPN‐B	2	79.5, 31.3	n.a.	n.a., II	121.1	Alive
14	F	58.6	Posterior fossa	II	PF‐EPN‐B	1	30.9	local	II	30.9	Alive
15	F	62.7	Posterior fossa	II	PF‐EPN‐B	1	39.7	local	II	39.7	Alive
16	M	0.4	Posterior fossa	III	none	1	5.3	local	III	20.6	Alive
17	M	18.4	Posterior fossa	II	none	1	118.7	local	III	119.7	Alive
18	M	13.0	Spinal	III	n.a.	1	25.0	local	III	25.0	Alive
19	F	21.3	Spinal	II	SP‐EPN‐MYCN	2	5.3, 1.0	local, metastatic	II, III	19.0	Alive
20	F	23.4	Spinal	I	n.a.	1	18.5	local	I	18.5	Alive
21	M	44.1	Spinal	III	SP‐MPE	1	70.8	local	III	70.8	Alive
22	F	45.0	Spinal	II	n.a.	1	96.0	local	II	96.0	Alive
23	F	51.7	Spinal	II	n.a.	1	53.7	local	II	69.9	Alive
24	F	59.8	Spinal	I	n.a.	1	8.1	local	I	8.1	Alive

For case #4, case #16 and case #17, DNA methylation was analyzed, but did not match to a known molecular subtype. n.a. = not available.

### Histomorphological and immunohistochemical evaluation

Using light microscopy, we scored cell density, necrosis and microvascular proliferation on H&E‐stained sections on a scale from 1 to 3 (cell density) and from 0 to 3 (necrosis and vessel proliferation) for primary (n = 12) and corresponding relapsed tumors (n = 21). Furthermore, we compared Ki67 (n = 14), H3K27me3 (n = 19) and CXorf67 immunohistochemistry (n = 15) between ependymoma primary and corresponding relapsed tumors. Ki67 was scored as percentage of positively stained tumor cells. Non‐tumor cells in the microenvironment including endothelial cells and immune cells served as internal positive controls for H3K27me3 immunohistochemistry. An unpaired *t*‐test was used for statistical analysis of the collected scores.

### Data

A public dataset was used to investigate methylation profiles of both primary and relapsed ependymal tumors from n = 45 patients (25) (GEO Accession number: GSE65362). Data of five molecular subgroups were available in this dataset: ST‐EPN‐RELA (n = 11), PF‐SE (n = 2), PF‐EPN‐A (n = 23), PF‐EPN‐B (n = 7) and SP‐MPE (n = 2). There were no data of tumors of the remaining four molecular subgroups ST‐SE, ST‐EPN‐YAP1, SP‐SE and SP‐EPN. In this experimental setup, Illumina HumanMethylation450 BeadChips were used (25).

### Data processing

The DNA methylation dataset (GSE65362) contains Beta‐values, the ratio of the methylated probe intensity and the overall intensity. To perform statistical analyses, these values were converted into M‐Values, that is, the log2 ratio of the intensities of methylated probe vs. unmethylated probe (5).

### Copy number variation analysis

For copy number variation (CNV) analysis, the Bioconductor package ChAMP was used (34). It used the HumanMethylation450 data to identify copy number alterations (CNA) by utilizing the intensity values for each probe to count copy number (CN) and determine whether CNAs are present. Here, the CNAs were compared between all primary and relapsed tumor samples as well as for every subgroup separately. For visualization, a heatmap for all patients was generated by computing the log‐fold change of intensity values between primary and relapsed tumors of the top 1000 loci. Conumee plots for individual samples were generated using a free online classifier tool (www.molecularneuropathology.org) (4).

### Differential methylation

Differentially methylated probes (DMPs) were computed applying a linear model using the R/limma package. DMPs were determined for relapse tumors compared to primary tumors with paired samples for all patients and within each molecular subgroup.

### Methylation status of different epigenomic substructures

Analysis was performed as previously described (30). For each probe, we have computed the average Beta‐value across all primary tumors (n = 45) and across all relapse tumors (n = 48). The dataset contains in total 485 577 probes. After mapping the probes onto epigenomic substructures, we got the following number of probes for each substructure: CpG Island n = 150 254, N Shelve n = 24 844, N Shore n = 62 870, S Shelve n = 22 300, S Shore n = 49 197, undefined/unmapped n = 176 112. In the following, we excluded all undefined/unmapped probes from the analysis. Next, we compared such computed mean Beta‐values between all primary and relapse samples for each substructure. Then, we repeated this comparison for each subgroup individually. A two‐sided *t*‐test of Beta‐values was applied. Significance was defined as 0.05.

### Fractions of hypo‐ and hypermethylated CpGs

Analysis was performed as previously described (30). For assessing fractions of hypo‐ and hypermethylated CpGs, only significantly methylated CpGs with *P*‐values < 0.05 were used, revealing 58 505 significant probes. Here, we used the uncorrected *P*‐value as we did not compare the probes with each other and just searched for small *P*‐values without ranking. Out of 58 505 probes, we identified 21 746 hypomethylated and 36 759 hypermethylated probes, respectively. Subsequently, the fractions of the substructures were computed, revealing percent values. The same analysis was performed for each subgroup. CpGs with log‐fold changes < 0 were defined as hypomethylated and CpGs with log‐fold changes > 0 as hypermethylated.

## Results

**Figure 1:**
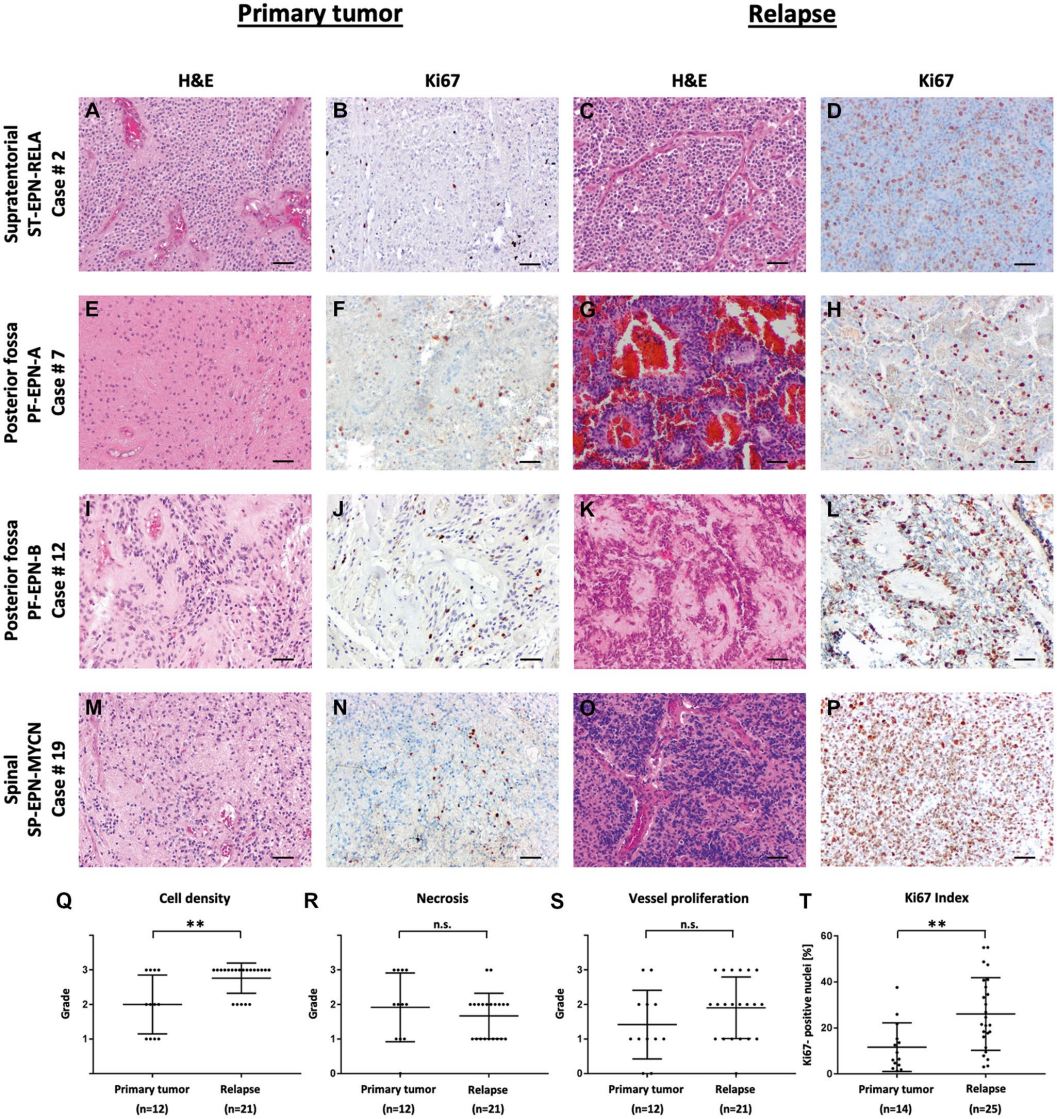
*Comparisons of morphology in primary and relapsed ependymomas*. Relapsed tumors demonstrate increase of cell density (Q, A, C, E, G, I, K, M, O) as well as intensified Ki67 labeling (T, B, D, F, H, J, L, N, P). Grades for cell density are as follows: 1 = low, 2 = medium, 3 = high. Grades for necrosis are as follows: 0 = absent, 1 = little, 2 = medium, 3 = plenty. Grades for vessel proliferation are as follows: 0 = absent, 1 = little, 2 = medium, 3 = plenty. Scale bars correspond to 50 µm.

We first aimed at elucidating potential morphological changes occurring during relapse/progression of ependymoma. To answer this question, we compared H&E‐stained sections of matched primary (n = 12) and relapsed ependymomas (n = 21) regarding typical features of malignancy (Figure 1A,C,E,G,I,K,M,O). Cell density was evaluated on a scale from 1 (low) to 3 (high). Similarly, the extent of necrosis and vessel proliferation were evaluated on a scale from 0 (absent) to 3 (plenty). We observed that cell density increased noticeably in relapsed tumors (Figure 1Q 2 ± 0.25 [mean ± SEM] for primary tumors vs. 2.76 ± 0.10 [mean ± SEM] for relapsed tumors, *P* = 0.0018, [*t*‐test]), whereas necrosis and vessel proliferation did not change significantly (Figure 1R, *P* = 0.39 [*t*‐test] and Figure 1S, *P* = 0.16 [*t*‐test]).

High tumor cell proliferation indexes are also a landmark of malignancy. In order to compare proliferation, Ki67 staining was performed, showing a significant increase in Ki67‐positive nuclei for relapsed (n = 25) in comparison to ependymoma primary tumor samples (n = 14) (Figure 1B,D,F,H,J,L,N,P,T, 11.62 ± 2.83 [mean ± SEM] for primary tumors vs. 26.05 ± 3.16 [mean ± SEM] for relapsed tumors, *P* = 0.0042 [*t*‐test]). This was true for all represented subgroups as shown in Figure 1 for ST‐EPN‐RELA (case #2), PF‐EPN‐A (case #7), PF‐EPN‐B (case #12) and SP‐EPN‐MYCN (case #19). Eight of the patients had more than one ependymoma relapse (Table 1) and material was available to score and compare Ki67 labeling for multiple samples from five such cases (Supporting Figure S1B). In general, proliferation increased over time in all patients (Supporting Figure S1). Together, we conclude from these results that ependymoma cells proliferate faster in a relapse situation in comparison to cells of the corresponding primary tumors.

**Figure 2:**
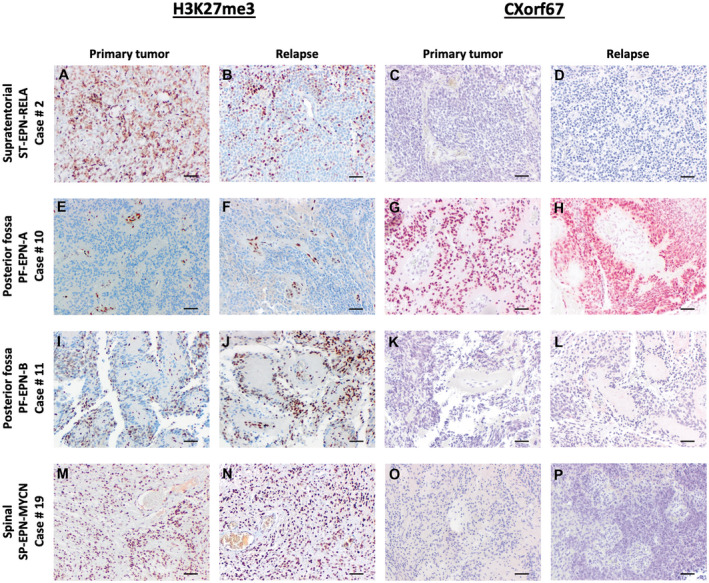
*Relapsed tumors show no change in H3K27me3 level and CXorf67 level*.

Loss of trimethylation at lysine 27 of the histone H3 gene and alterations in CXorf67 level, recently renamed into EZH Inhibitory Protein (EZHIP) (13), are important events in PF‐EPN‐A tumorigenesis (24, 26). By immunohistochemistry, we evaluated the association between the expression of these markers and ependymoma relapse by comparing all available H3K27me3‐ (n = 19) and CXorf67‐stained sections (n = 15) for the matched tumor samples. As expected, PF‐EPN‐A tumors showed a major loss of H3K27 trimethylation (Figure 2E,F), but stained positive for CXorf67 (Figure 2G‐H). In contrast, tumors of all other subgroups including ST‐EPN‐RELA, PF‐EPN‐B and spinal SP‐EPN‐MYCN ependymoma stained H3K27me3‐positive and CXorf67‐negative (Figure 2A‐D,I‐P). Most importantly, we did not observe any significant change in H3K27 trimethylation or CXorf67 expression levels between primary and relapsed ependymoma samples as shown exemplarily for four cases from different anatomical sites in Figure 2.

As a next step, we aimed at investigating CNA, differential DNA methylation as well as methylation status and fractions of hypo‐ and hypermethylated CpGs within different epigenomic substructures in primary and relapsed ependymomas. First, we exemplarily analyzed three cases from our cohort (Table 1). CNV plots of primary and relapsed ependymoma samples (cases #2, #15) and two relapsed ependymoma samples from the same case (case #10) turned out to be very similar (Supporting Figure S2A). Also, based on their global DNA methylation, the same above‐mentioned three cases clustered together in a t‐SNE plot, suggesting a similar global DNA methylation profile (Supporting Figure S2B). Finally, Nmyc overexpression was present in both relapsed tumor samples of case #19, a SP‐EPN‐MYCN ependymoma (Supporting Figure S2C).

**Figure 3:**
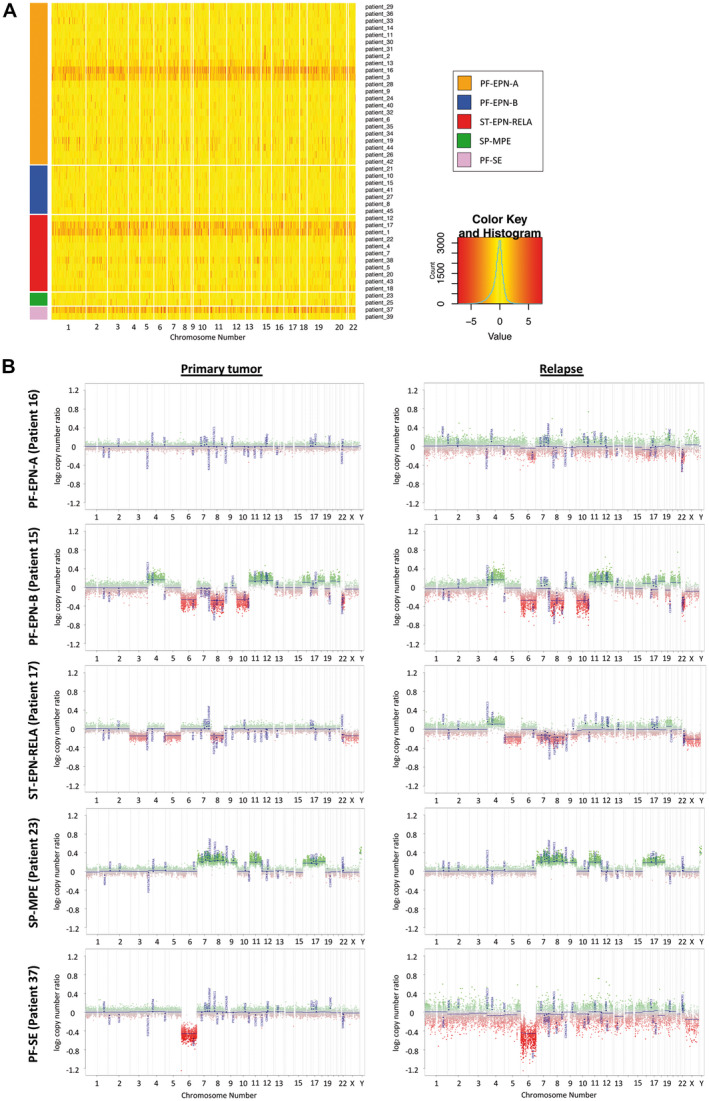
*Copy number variations at ependymoma relapse remain relatively stable*. **A.** Heatmap of copy number variation of all patients showing logarithmic fold changes of intensity values between ependymoma relapsed tumor samples (n = 48) compared to primary tumor samples (n = 45) of the top 1000 loci. Patients are grouped by five molecular subgroups. **B.** Representative copy number variation plots for pairs of samples are shown for each of the five represented subgroups. Raw data were obtained from Pajtler et al. (25)

In order to analyze these aspects in more depth, we used public DNA methylation data from 45 patients (25) (GSE65362). CNVs at ependymoma relapse compared to the corresponding primary tumor remained relatively stable as shown in a heatmap for each patient individually (n = 45), although some of the patients seemed to demonstrate more changes in CNV by comparing relapsed and primary ependymoma samples than the rest (Figure 3A). Representative CNV plots for pairs of samples from each of the five represented subgroups in this series are shown in Figure 3B.

**Figure 4:**
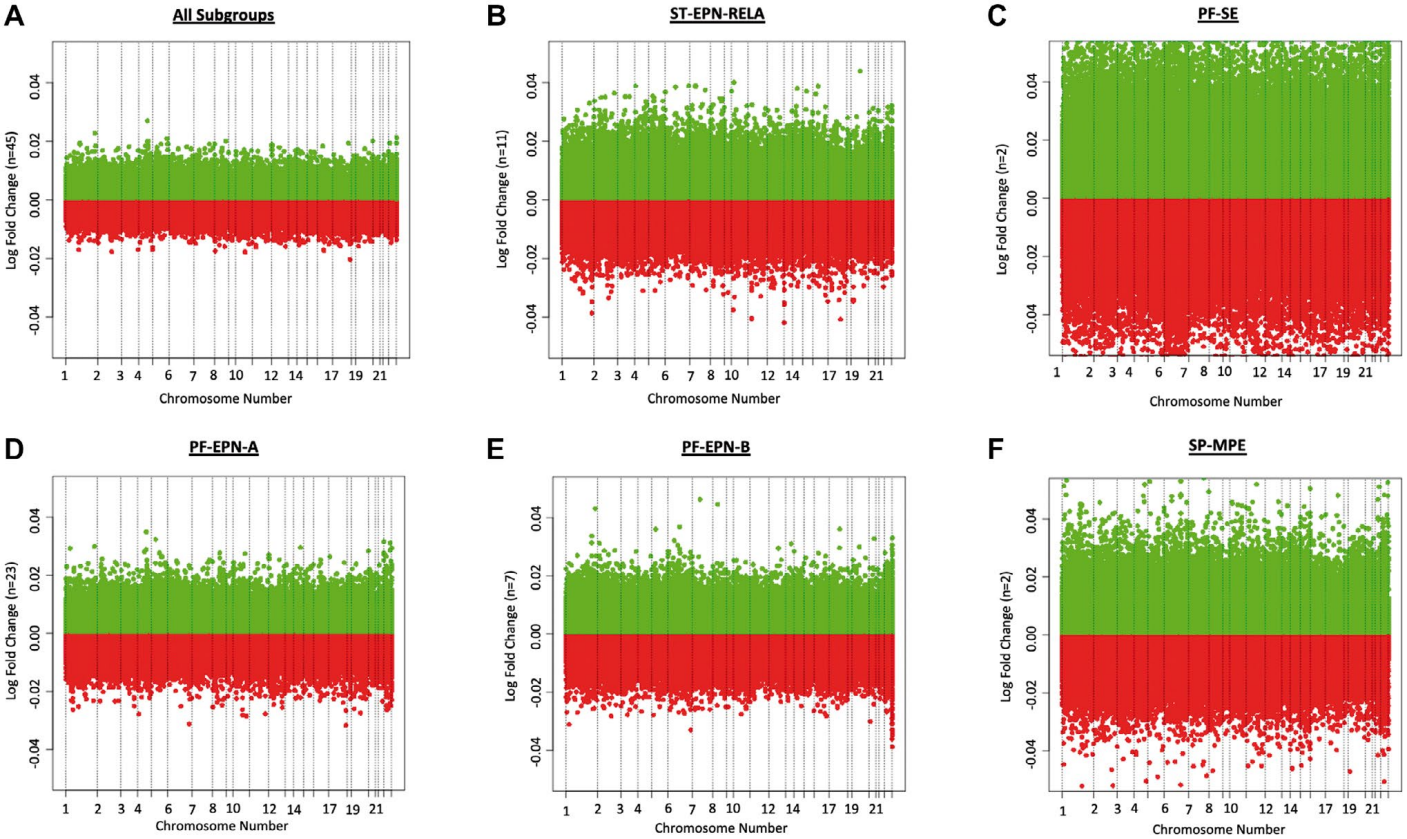
*Log–transformed ratios of methylation difference in ependymoma relapsed tumor samples (n = 48) compared to primary tumor samples (n = 45)*. The diagrams are shown for the whole cohort as well as separately for each of the five represented subgroups. Neither in the whole cohort nor in one of the represented subgroups significant differentially methylated regions were found. Raw data were obtained from Pajtler et al. (25)

Differential DNA methylation within the chromosomes was analyzed for the whole cohort (n = 45) as well as for each of the represented subgroups ST‐EPN‐RELA (n = 11), PF‐SE (n = 2), PF‐EPN‐A (n = 23), PF‐EPN‐B (n = 7) and SP‐MPE (n = 2). No significant difference with respect to the overall DNA methylation was detected after multiple testing for ependymoma relapse compared to primary tumor samples, neither for the whole cohort (Figure 4A) nor for any of the above‐mentioned molecular subgroups (Figure 4B‐F). Figure 4 shows the log‐transformed ratios of DNA methylation differences for each probe in ependymoma relapse compared to primary tumor samples.

**Figure 5:**
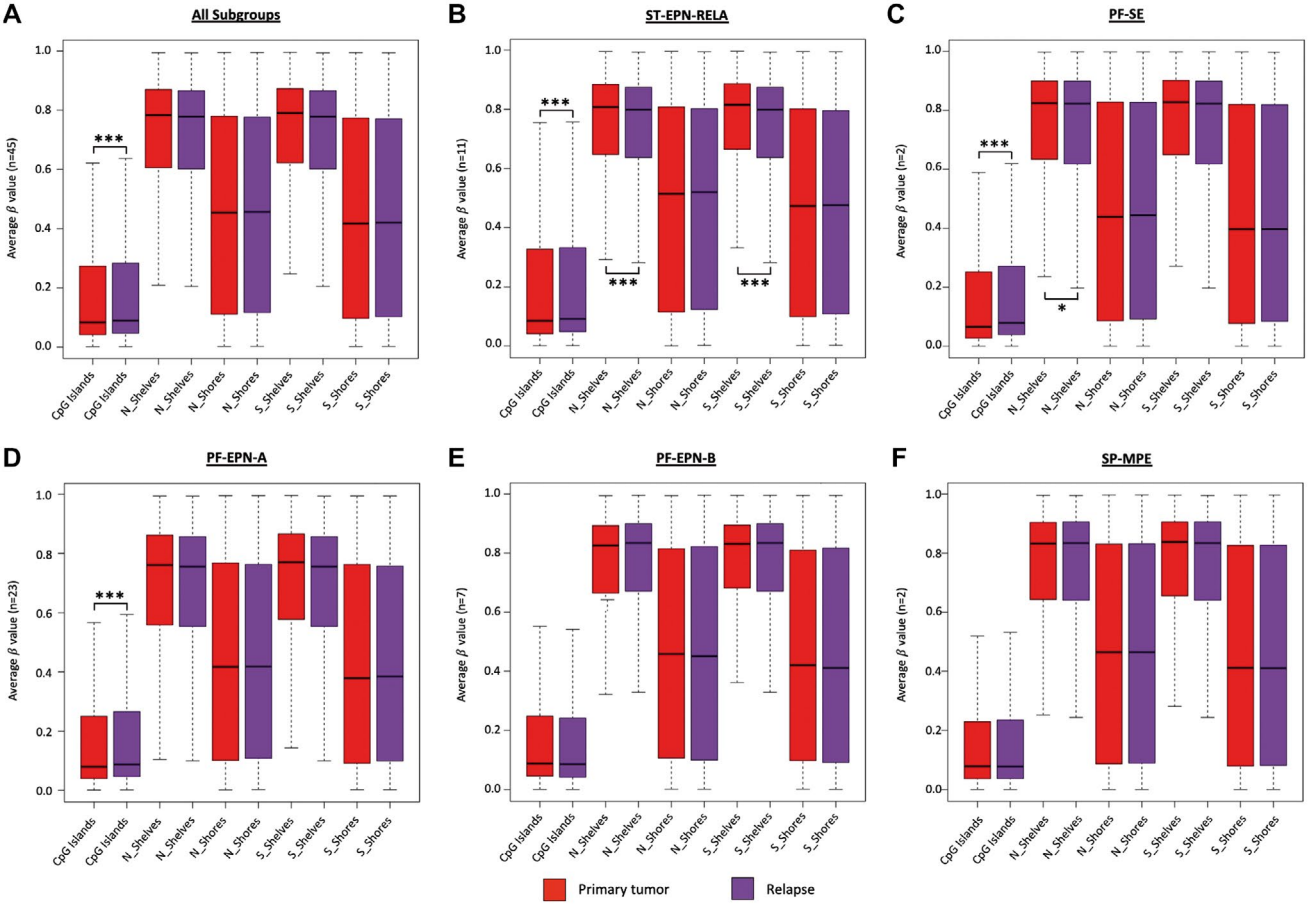
*Methylation status of the different epigenomic substructures in ependymoma relapsed tumor samples (n = 48) compared to primary tumor samples (n = 45)*. The box plots are shown for the whole cohort as well as separately for each of the represented subgroups. Significant hyper‐ or hypomethylation was found for CpG Islands in the whole cohort as well as in ST‐EPN‐RELA, PF‐SE and PF‐EPN‐A tumors, N_Shelves and S_Shelves in ST‐EPN‐RELA and N_Shelves in PF‐SE. Raw data were obtained from Pajtler et al. (25)

Next, we investigated the methylation status of several different epigenomic substructures, including CpG islands, shelves and shores for both primary and relapse ependymoma samples. While the regions located up to 2 kb from CpG islands are termed shores (14), the 2 kb regions flanking the shores up‐ and downstream are termed shelves (3). Our analysis revealed significantly higher methylation values in relapsed compared to primary tumor samples within CpG islands when looking at the whole cohort (Figure 5A, *P* = 6.04e‐6) as well as at ST‐EPN‐RELA (Figure 5B, *P* = 1.17e‐6), PF‐SE (Figure 5C, *P* = 2.77e‐14) and PF‐EPN‐A (Figure 5D, *P* = 1.15e‐9). Moreover, N Shelves were significantly less methylated in ST‐EPN‐RELA (Figure 5B, *P* = 1.20e‐4) and PF‐SE recurrent ependymomas (Figure 5C, *P* = 0.04) as were S Shelves in ST‐EPN‐RELA relapsed ependymomas (Figure 5B, *P* = 1.22e‐4). Thus, the DNA methylation values of epigenomic substructures, especially CpG islands, may change during relapse of ependymoma.

**Figure 6:**
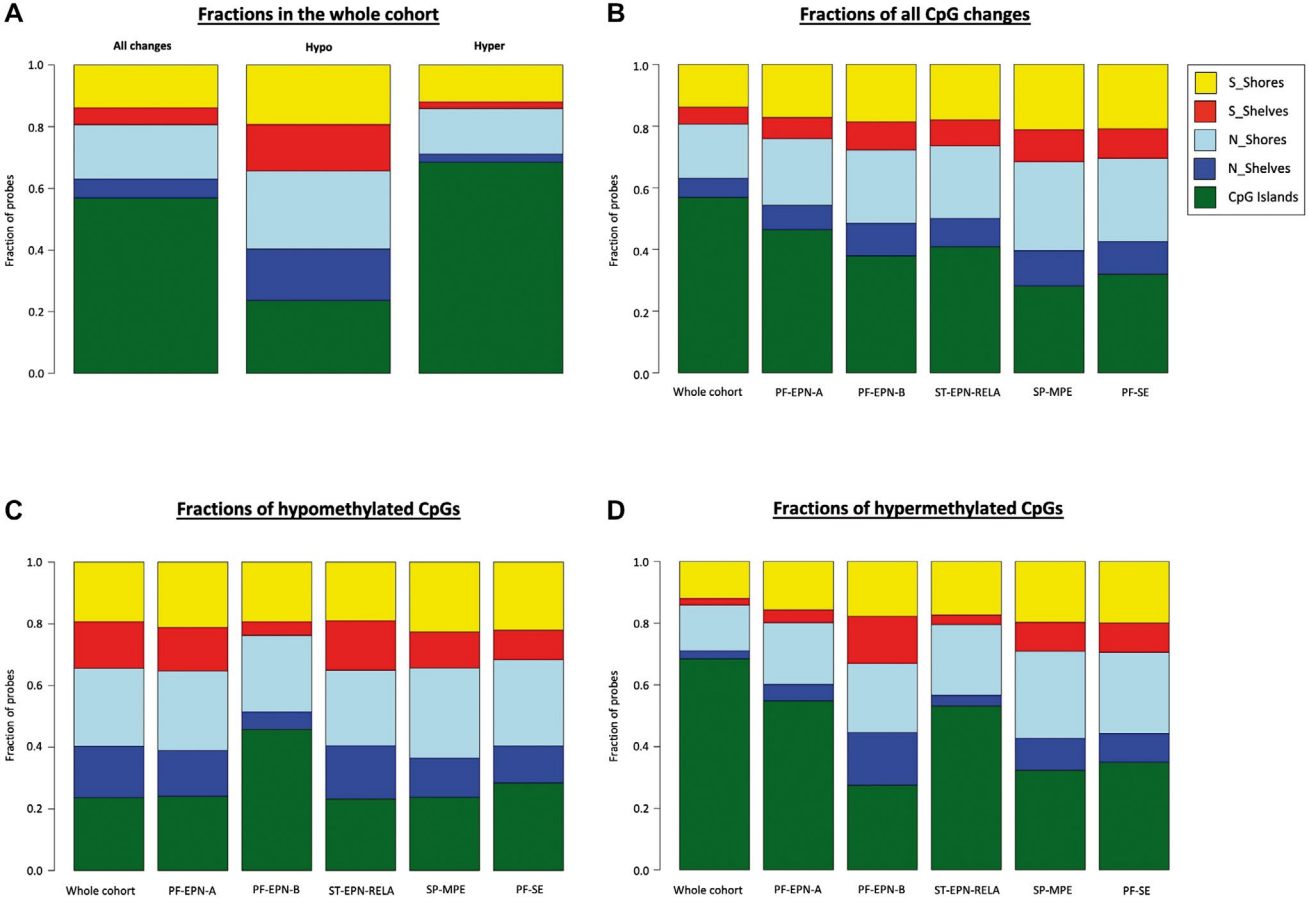
*Fractions of hypo‐ and hypermethylated CpGs in ependymoma relapsed tumor samples (n = 48) in comparison to primary tumor samples (n = 45) within different epigenomic substructures*. Diagram A shows the fractions of all changes, meaning hypo‐ and hypermethylated CpGs summed up, and of hypo‐ and hypermethylated CpGs, respectively, for the whole cohort. Diagrams B, C and D show the fractions of all CpG changes, hypo‐ and hypermethylated CpGs, respectively, for the whole cohort as well as separately for each of the five represented subgroups. Raw data were obtained from Pajtler et al. (25)

To address the question how these changes in DNA methylation look like in more detail and whether unchanged overall methylation values resulted from hypo‐ and hypermethylation at different sites, we particularly looked at hypo‐ and hypermethylation in distinct subgroups and genomic regions. As shown in Figure 6A, DNA methylation changes mainly affect the CpG islands. Moreover, in line with the results shown in Figure 5, hypermethylation mainly occurs in CpG islands, whereas hypomethylation similarly happens in all regions (Figure 6A). Regarding the distinct ependymoma subgroups, CpG changes appear to be similarly distributed over the genomic regions with most changes in the CpG islands and least changes in shelves for most of the subgroups (Figure 6B). Finally, each subgroup shows a different pattern of fractions of hypo‐ or hypermethylated probes in the different epigenomic substructures (Figure 6C‐D), indicating that relapse of ependymoma proceeds differently on epigenetic level for every subgroup.

## Discussion

In our study, we focused on the investigation of histomorphological, immunohistochemical, chromosomal, and epigenetic differences during ependymoma relapse. We compared ependymoma primary and relapsed tumor samples of 24 patients histomorphologically and immunohistochemically and used public methylation data of 45 patients with primary and relapsed ependymal tumors (25) for chromosomal and epigenetic comparison. By hierarchical clustering of DNA methylation profiles, Pajtler et al. used their data to demonstrate that molecular subgroups remain stable at ependymoma recurrence (25), which we exemplarily confirmed for three of our cases (Supporting Figure S2). However, a comparison of primary and relapsed ependymomas on chromosomal and epigenetic level was not previously conducted, which is what we used the data for in the current work.

In contradiction to Pajtler et al. (25), an earlier study reported subgroup switching between the two posterior fossa groups A and B. More precisely, an analysis of seven group A and seven group B paired samples revealed switching of three group A and one group B posterior fossa ependymoma to the other subgroup at first recurrence, respectively. Here, the transcriptionally distinct subgroups were identified by hierarchical clustering of the top 5% of differentially expressed genes (883 genes in total) (12). However, more reliable classification tools for distinguishing the two posterior fossa ependymoma groups A and B, namely methylation profiling (25) and H3K27me3 immunohistochemistry, which has a sensitivity of 99% and a specificity of 100% in segregating PF‐EPN‐A from PF‐EPN‐B tumors (26), were discovered in the meantime. For this reason, we assume that the reported changes in subgroup rather reflected an incorrect classification of some of the cases either at diagnosis or at recurrence than a real subgroup switch. Indeed, the authors failed to show an effect of subgroup switching on clinical outcome, which one would expect given the clearly distinct clinical features of both subgroups (25). Admittedly, the small number of cases made it difficult to prove such a point. Still, consensus nonnegative matrix factorization in the earlier paper only demonstrated a concordance of 92.8% and 76.3% for group A and B posterior fossa ependymomas, respectively (12), strongly supporting our hypothesis.

It was reported previously that histological features such as cellularity, necrosis, vascular proliferation, as well as mitotic count taken together are associated with a dismal outcome in ependymoma. High proliferation indices were encountered in ependymal tumors that behaved in a more aggressive manner (28). We found a significant increase in cell density and proliferation in relapsed tumors compared to the corresponding primary tumors, hypothesizing that relapsed ependymomas behave more aggressively. However, we did not detect a significant change in the degree of vessel proliferation or necrosis, which could be attributed to the relatively small sample size.

It has been shown that H3K27me3 staining is globally and exclusively reduced in PFA ependymomas (26). Furthermore, a recent study found high levels of CXorf67 expression in almost all PFA subtypes except PFA‐1f, a subtype that shows high frequencies of H3K27M mutations (24). Although only three out of six PFA ependymomas in this study were tested for the histone H3K27M mutation, these cases turned out to be negative, being in line with our observation that all PFA ependymoma cases stained negative for H3K27me3 and positive for CXorf67 in contrast to all other subgroups (Figure 2).

We did not observe any significant changes in H3K27me3 and CXorf67 during relapse in any of the paired samples. This is in agreement with the assumption that the molecular subgroup, based on DNA methylation profiling, remains stable at disease recurrence (25). Still, we did find some focal intratumoral differences within samples, especially regarding H3K27me3 staining, which probably reflect the high histological variability and intratumoral heterogeneity of ependymomas (7).

It should be noted that the public methylation data (25) we used to look for chromosomal and epigenetic differences do not represent all of the known molecular ependymoma subgroups. So, further research is needed with respect to the four molecular subgroups ST‐SE, ST‐EPN‐YAP1, SP‐SE and SP‐EPN, that are not represented in this study, as well as for the additional molecular subgroups and subtypes of ependymoma that have subsequently been described (8, 24).

CNV analysis did not reveal any significant differences between primary and relapsed ependymoma samples, which is in line with a former study using SNPs to investigate CNA in 11 matched primary and recurrent posterior fossa ependymomas. This study also proposed that transcriptomic and microenvironmental, rather than genomic changes, might be of relevance at recurrence. In particular, the authors referred to differences in the immunophenotype of group A and B posterior fossa ependymomas in general as well as within the respective subgroups at diagnosis and recurrence (12). However, Puget et al. (29) found increased genomic instability in a cohort of 26 relapsed tumors compared to 33 primary ependymomas. Among other imbalances, including gain of 1q and loss of 6q, gain of 9q34 was found to occur more often in relapsed ependymal tumors. Moreover, gains of 9q33 and 9q34 were significantly associated with tumor recurrence, age older than 3 years and posterior fossa location. It was suggested that two potential oncogenes, NOTCH1 and Tenascin‐C, located on 9qter and found to be overexpressed might play a role in ependymoma progression. Additionally, a small number of posterior fossa ependymomas with increased genomic instability on chromosome 9, but also on other chromosomes at relapse were reported previously (9, 32). Regarding these reported observations for individual patients, we assume, in light of our findings (Figure 3), that they cannot be generalized for a specific subgroup or for ependymomas at relapse.

Although overall DNA methylation did not change (Figure 4), CpG island methylation increased significantly in relapsed ependymomas for all represented subgroups except for PF‐EPN‐B and SP‐MPE. In contrast, relapsed compared to primary ependymomas demonstrated significant hypomethylation of N Shelves in the subgroups ST‐EPN‐RELA and PF‐SE and of S Shelves in ST‐EPN‐RELA (Figure 5). While hypermethylation of CpG islands is widely considered to be characteristic for cancers (1, 2, 6), not much is known about methylation of CpG islands in relapsed tumors. Increased methylation of certain genes has been reported for AML at relapse (17) and aberrant DNA methylation for five genomic loci was shown for a cohort of 25 relapsed ependymomas before (36). Hypermethylation in general seems to occur more often in CpG islands, whereas hypomethylation rather affects other areas, including shores and shelves. This was demonstrated for sessile serrated adenomas with dysplasia, which can progress to colorectal cancer, compared to ordinary sessile serrated adenomas (18), for breast cancer compared to normal breast tissue (15) and for hepatocellular carcinoma, associated with hepatitis C virus or chronic alcohol abuse, compared to normal liver tissue (11, 21, 33). Furthermore, a recent study compared primary colorectal cancer with non‐matched liver metastases. Hypermethylated DMRs occurred more often in CpG islands than in shelves or shores in both primary colorectal cancers and liver metastases compared to normal colon mucosa, whereas hypomethylated DMRs where distributed equally among CpG islands, shores and shelves. Interestingly, hypomethylation seemed to occur continually in metastatic lesions, whereas hypermethylation remained similar between primary colorectal cancer and liver metastasis. Thus, it was speculated that hypomethylation in liver metastases is linked to drug resistance (22). As for our study, the relevance and impact of CpG island hypermethylation and hypomethylation of shelves in the relapse situation remain unclear and need to be further elucidated.

In conclusion, relapsed ependymomas demonstrate increased features of malignancy, such as increased cell density and high proliferation rate. Although we did find slight changes in the epigenome of relapsed tumors, namely CpG island hypermethylation, these findings cannot fully explain the altered tumor morphology. Thus, further research is needed to identify the molecular mechanisms of ependymoma progression.

## Conflict of Interest

None.

## Supporting information

Fig S1
**Figure S1.** Histomorphology worsens over time, despite some exceptions. Considering the small number of cases with multiple relapses no general conclusion can be drawn. **A. **Course of histomorphological changes over three relapses shown exemplarily for one case. Relapse 2 demonstrates massive necrosis. Scale bars correspond to 50 µm. B. Ki67 index in five cases with multiple relapses. Cases belong to subgroups ST‐EPN‐RELA (#2), PF‐EPN‐A (#10), PF‐EPN‐B (#11, #12) and SP‐EPN‐MYCN (#19), respectively. The X‐axis shows the sample number. Within the sample number, the first number indicates the case number and the second number indicates the number of relapse. 0 refers to the respective primary tumor. The Y‐axis represents the percentage of Ki67‐positive nuclei.

Fig S2
**Figure S2.** Ependymoma relapsed tumor samples do not change their molecular subgroup compared to primary tumor samples. **A. **Copy number variations remain relatively stable between primary and relapsed tumor samples as well as between multiple relapsed tumor samples, exemplarily shown for two samples of three cases, respectively. **B. **Samples from the same three cases as in A* *cluster together as shown in the t‐SNE plot. **C. **Nmyc overexpression is present in both relapsed tumors of a SP‐EPNMYCN ependymoma.

## Data Availability

A public dataset was used to investigate methylation profiles of both primary and relapsed ependymal tumors from n = 45 patients (25) (GEO Accession number: GSE65362).

## References

[1] Baylin SB , Jones PA (2011) A decade of exploring the cancer epigenome ‐ biological and translational implications. Nat Rev Cancer 11:726–734. 21941284 10.1038/nrc3130 PMC3307543

[2] Berman BP , Weisenberger DJ , Aman JF , Hinoue T , Ramjan Z , Liu Y , *et al*. (2011) Regions of focal DNA hypermethylation and long‐range hypomethylation in colorectal cancer coincide with nuclear lamina‐associated domains. Nat Genet 44:40–46. 22120008 10.1038/ng.969 PMC4309644

[3] Bibikova M , Barnes B , Tsan C , Ho V , Klotzle B , Le JM *et al* (2011) High density DNA methylation array with single CpG site resolution. Genomics 98:288–295. 21839163 10.1016/j.ygeno.2011.07.007

[4] Capper D , Jones DTW , Sill M , Hovestadt V , Schrimpf D , Sturm D *et al* (2018) DNA methylation‐based classification of central nervous system tumours. Nature 555:469–474. 29539639 10.1038/nature26000 PMC6093218

[5] Du P , Zhang X , Huang CC , Jafari N , Kibbe WA , Hou L , Lin SM (2010) Comparison of Beta‐value and M‐value methods for quantifying methylation levels by microarray analysis. BMC Bioinformatics 11:587. 21118553 10.1186/1471-2105-11-587 PMC3012676

[6] Ehrlich M (2019) DNA hypermethylation in disease: mechanisms and clinical relevance. Epigenetics 14:1141–1163. 31284823 10.1080/15592294.2019.1638701 PMC6791695

[7] Ellison DW , Kocak M , Figarella‐Branger D , Felice G , Catherine G , Pietsch T *et al* (2011) Histopathological grading of pediatric ependymoma: reproducibility and clinical relevance in European trial cohorts. J Negat Results Biomed 10:7. 21627842 10.1186/1477-5751-10-7 PMC3117833

[8] Ghasemi DR , Sill M , Okonechnikov K , Korshunov A , Yip S , Schutz PW *et al* (2019) MYCN amplification drives an aggressive form of spinal ependymoma. Acta Neuropathol 138:1075–1089. 31414211 10.1007/s00401-019-02056-2 PMC6851394

[9] Grill J , Avet‐Loiseau H , Lellouch‐Tubiana A , Sevenet N , Terrier‐Lacombe MJ , Venuat AM *et al* (2002) Comparative genomic hybridization detects specific cytogenetic abnormalities in pediatric ependymomas and choroid plexus papillomas. Cancer Genet Cytogenet 136:121–125. 12237235 10.1016/s0165-4608(02)00516-2

[10] Hill RM , Kuijper S , Lindsey JC , Petrie K , Schwalbe EC , Barker K *et al* (2015) Combined MYC and P53 defects emerge at medulloblastoma relapse and define rapidly progressive, therapeutically targetable disease. Cancer Cell 27:72–84. 25533335 10.1016/j.ccell.2014.11.002 PMC4297293

[11] Hlady RA , Tiedemann RL , Puszyk W , Zendejas I , Roberts LR , Choi JH *et al* (2014) Epigenetic signatures of alcohol abuse and hepatitis infection during human hepatocarcinogenesis. Oncotarget 5:9425–9443. 25294808 10.18632/oncotarget.2444 PMC4253444

[12] Hoffman LM , Donson AM , Nakachi I , Griesinger AM , Birks DK , Amani V *et al* (2014) Molecular sub‐group‐specific immunophenotypic changes are associated with outcome in recurrent posterior fossa ependymoma. Acta Neuropathol 127:731–745. 24240813 10.1007/s00401-013-1212-8 PMC3988227

[13] Hubner JM , Muller T , Papageorgiou DN , Mauermann M , Krijgsveld J , Russell RB *et al* (2019) EZHIP / CXorf67 mimics K27M mutated oncohistones and functions as an intrinsic inhibitor of PRC2 function in aggressive posterior fossa ependymoma. Neuro Oncol 21:878–889. 30923826 10.1093/neuonc/noz058 PMC6620627

[14] Irizarry RA , Ladd‐Acosta C , Wen B , Wu Z , Montano C , Onyango P *et al* (2009) The human colon cancer methylome shows similar hypo‐ and hypermethylation at conserved tissue‐specific CpG island shores. Nat Genet 41:178–186. 19151715 10.1038/ng.298 PMC2729128

[15] Jin W , Li QZ , Zuo YC , Cao YN , Zhang LQ , Hou R , Su WX (2019) Relationship between DNA methylation in key region and the differential expressions of genes in human breast tumor tissue. DNA Cell Biol 38:49–62. 30346835 10.1089/dna.2018.4276

[16] Klughammer J , Kiesel B , Roetzer T , Fortelny N , Nemc A , Nenning KH *et al* (2018) The DNA methylation landscape of glioblastoma disease progression shows extensive heterogeneity in time and space. Nat Med 24:1611–1624. 30150718 10.1038/s41591-018-0156-x PMC6181207

[17] Kroeger H , Jelinek J , Estecio MR , He R , Kondo K , Chung W *et al* (2008) Aberrant CpG island methylation in acute myeloid leukemia is accentuated at relapse. Blood 112:1366–1373. 18523155 10.1182/blood-2007-11-126227 PMC2515110

[18] Liu C , Fennell LJ , Bettington ML , Walker NI , Dwine J , Leggett BA , Whitehall VLJ (2019) DNA methylation changes that precede onset of dysplasia in advanced sessile serrated adenomas. Clin Epigenetics 11:90. 31200767 10.1186/s13148-019-0691-4 PMC6570920

[19] Louis DN , Ohgaki H , Wiestler OD , Cavenee WK , Ellison DW , Figarella‐Branger D *et al* (2016) WHO classification of tumours of the central nervous system, revised 4th edn. Lyon: International Agency for Research on Cancer.

[20] Marinoff AE , Ma C , Guo D , Snuderl M , Wright KD , Manley PE *et al* (2017) Rethinking childhood ependymoma: a retrospective, multi‐center analysis reveals poor long‐term overall survival. J Neurooncol 135:201–211. 28733870 10.1007/s11060-017-2568-8 PMC5658456

[21] Minarovits J , Demcsák A , Banati F , Niller HH (2016) Epigenetic dysregulation in virus‐associated neoplasms. Adv Exp Med Biol 879:71–90. 26659264 10.1007/978-3-319-24738-0_4

[22] Orjuela S , Menigatti M , Schraml P , Kambakamba P , Robinson MD , Marra G (2020) The DNA hypermethylation phenotype of colorectal cancer liver metastases resembles that of the primary colorectal cancers. BMC Cancer 20:290. 32252665 10.1186/s12885-020-06777-6 PMC7137338

[23] Pajtler KW , Mack SC , Ramaswamy V , Smith CA , Witt H , Smith A *et al* (2017) The current consensus on the clinical management of intracranial ependymoma and its distinct molecular variants. Acta Neuropathol 133:5–12. 27858204 10.1007/s00401-016-1643-0 PMC5209402

[24] Pajtler KW , Wen J , Sill M , Lin T , Orisme W , Tang B *et al* (2018) Molecular heterogeneity and CXorf67 alterations in posterior fossa group A (PFA) ependymomas. Acta Neuropathol 136:211–226. 29909548 10.1007/s00401-018-1877-0 PMC6105278

[25] Pajtler KW , Witt H , Sill M , Jones DT , Hovestadt V , Kratochwil F *et al* (2015) Molecular classification of ependymal tumors across all cns compartments, histopathological grades, and age groups. Cancer Cell 27:728–743. 25965575 10.1016/j.ccell.2015.04.002 PMC4712639

[26] Panwalkar P , Clark J , Ramaswamy V , Hawes D , Yang F , Dunham C *et al* (2017) Immunohistochemical analysis of H3K27me3 demonstrates global reduction in group‐A childhood posterior fossa ependymoma and is a powerful predictor of outcome. Acta Neuropathol 134:705–714. 28733933 10.1007/s00401-017-1752-4 PMC5647236

[27] Parker M , Mohankumar KM , Punchihewa C , Weinlich R , Dalton JD , Li Y *et al* (2014) C11orf95‐RELA fusions drive oncogenic NF‐kappaB signalling in ependymoma. Nature 506:451–455. 24553141 10.1038/nature13109 PMC4050669

[28] Prayson RA (1999) Clinicopathologic study of 61 patients with ependymoma including MIB‐1 immunohistochemistry. Ann Diagn Pathol 3:11–18. 9990108 10.1016/s1092-9134(99)80004-5

[29] Puget S , Grill J , Valent A , Bieche I , Dantas‐Barbosa C , Kauffmann A *et al* (2009) Candidate genes on chromosome 9q33‐34 involved in the progression of childhood ependymomas. J Clin Oncol 27:1884–1892. 19289631 10.1200/JCO.2007.15.4195

[30] Rodriguez‐Paredes M , Bormann F , Raddatz G , Gutekunst J , Lucena‐Porcel C , Kohler F *et al* (2018) Methylation profiling identifies two subclasses of squamous cell carcinoma related to distinct cells of origin. Nat Commun 9:577. 29422656 10.1038/s41467-018-03025-1 PMC5805678

[31] Sabel M , Fleischhack G , Tippelt S , Gustafsson G , Doz F , Kortmann R *et al* (2016) Relapse patterns and outcome after relapse in standard risk medulloblastoma: a report from the HIT‐SIOP‐PNET4 study. J Neurooncol 129:515–524. 27423645 10.1007/s11060-016-2202-1 PMC5020107

[32] Schneider D , Monoranu CM , Huang B , Rutkowski S , Gerber NU , Krauss J *et al* (2009) Pediatric supratentorial ependymomas show more frequent deletions on chromosome 9 than infratentorial ependymomas: a microsatellite analysis. Cancer Genet Cytogenet 191:90–96. 19446744 10.1016/j.cancergencyto.2009.02.010

[33] Shen J , Wang S , Zhang YJ , Wu HC , Kibriya MG , Jasmine F *et al* (2013) Exploring genome‐wide DNA methylation profiles altered in hepatocellular carcinoma using Infinium HumanMethylation 450 BeadChips. Epigenetics 8:34–43. 23208076 10.4161/epi.23062 PMC3549879

[34] Tian Y , Morris TJ , Webster AP , Yang Z , Beck S , Feber A , Teschendorff AE (2017) ChAMP: updated methylation analysis pipeline for Illumina BeadChips. Bioinformatics 33:3982–3984. 28961746 10.1093/bioinformatics/btx513 PMC5860089

[35] Villano JL , Parker CK , Dolecek TA (2013) Descriptive epidemiology of ependymal tumours in the United States. Br J Cancer 108:2367–2371. 23660944 10.1038/bjc.2013.221 PMC3681017

[36] Xie H , Wang M , Bonaldo Mde F , Rajaram V , Stellpflug W , Smith C *et al* (2010) Epigenomic analysis of Alu repeats in human ependymomas. Proc Natl Acad Sci U S A 107:6952–6957. 20351280 10.1073/pnas.0913836107 PMC2872440

