# Supplementary figures and images for: Ependymoma relapse goes along with a relatively stable epigenome, but a severely altered tumor morphology
Source: Brain Pathol. 2020 Jul 28;31(1):33–44. doi: 10.1111/bpa.12875 (PMC8018105; doi:10.1111/bpa.12875)

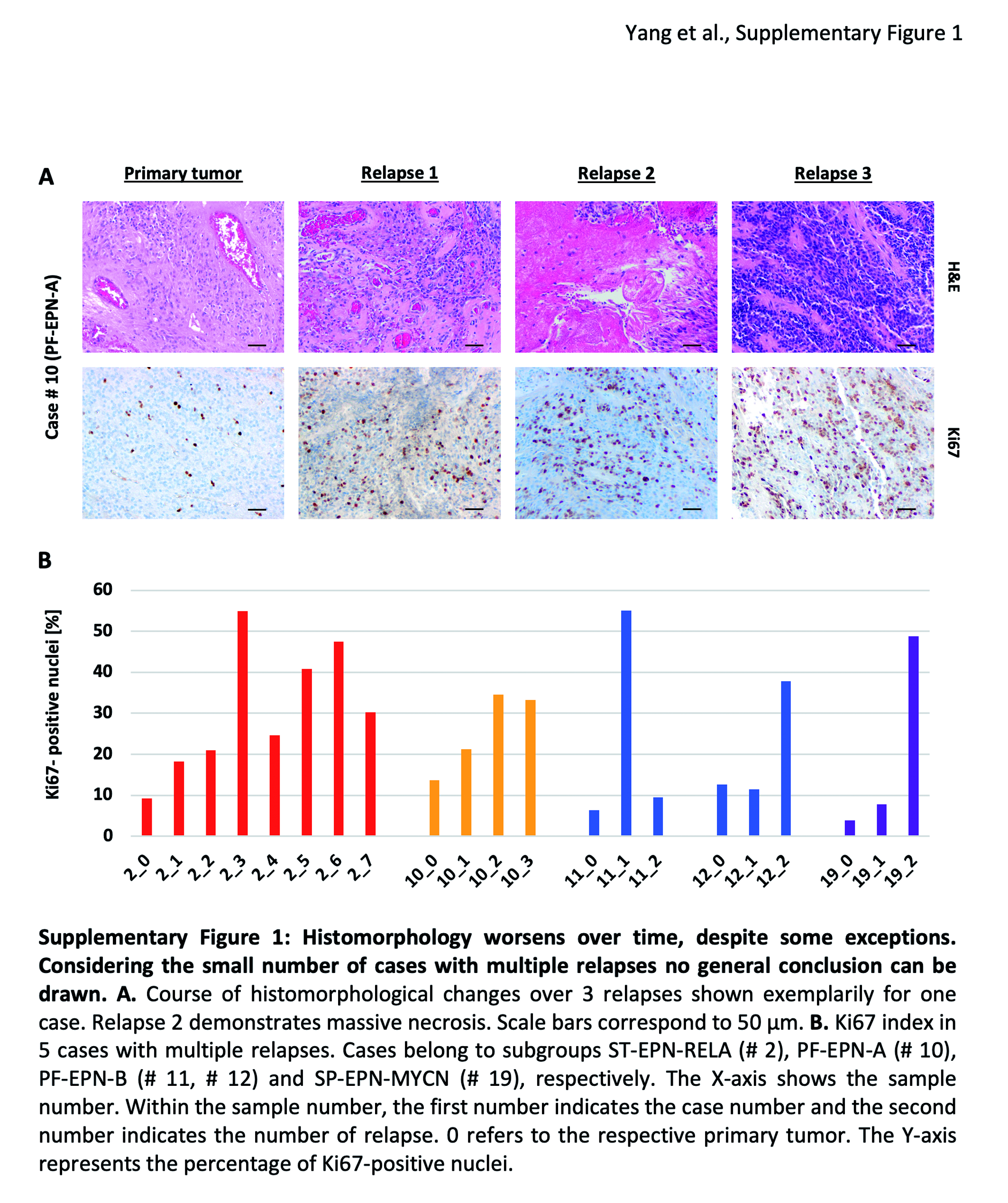

Supplement: Supplementary file 1 — Fig S1 Figure S1. Histomorphology worsens over time, despite some exceptions. Considering the small number of cases with multiple relapses no general conclusion can be drawn. A. Course of histomorphological changes over three relapses shown exemplarily for one case. Relapse 2 demonstrates massive necrosis. Scale bars correspond to 50 µm. B. Ki67 index in five cases with multiple relapses. Cases belong to subgroups ST‐EPN‐RELA (#2), PF‐EPN‐A (#10), PF‐EPN‐B (#11, #12) and SP‐EPN‐MYCN (#19), respectively. The X‐axis shows the sample number. Within the sample number, the first number indicates the case number and the second number indicates the number of relapse. 0 refers to the respective primary tumor. The Y‐axis represents the percentage of Ki67‐positive nuclei. [file BPA-31-33-s001.tif]

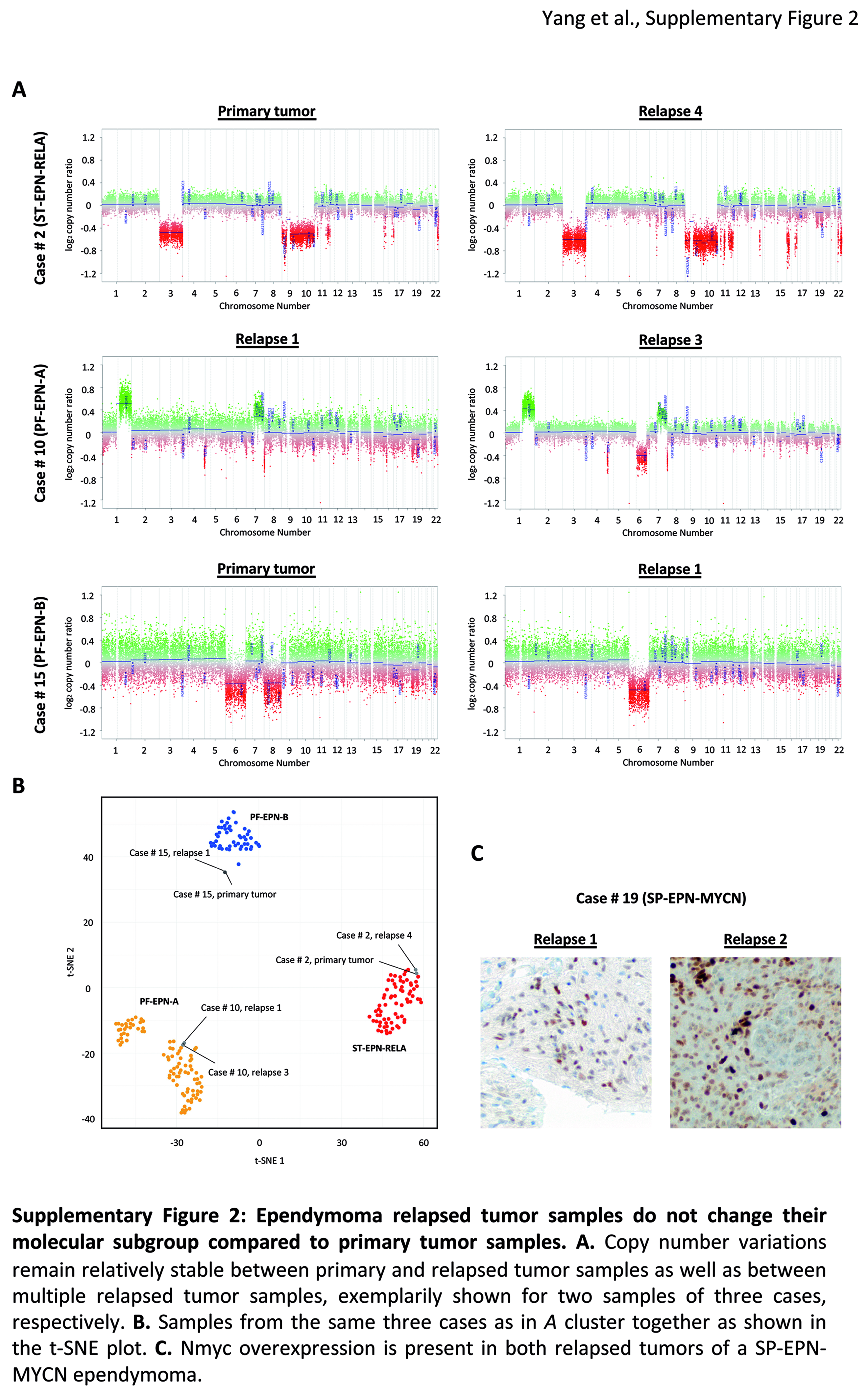

Supplement: Supplementary file 2 — Fig S2 Figure S2. Ependymoma relapsed tumor samples do not change their molecular subgroup compared to primary tumor samples. A. Copy number variations remain relatively stable between primary and relapsed tumor samples as well as between multiple relapsed tumor samples, exemplarily shown for two samples of three cases, respectively. B. Samples from the same three cases as in A cluster together as shown in the t‐SNE plot. C. Nmyc overexpression is present in both relapsed tumors of a SP‐EPNMYCN ependymoma. [file BPA-31-33-s002.tif]
